# Small Supernumerary Marker Chromosome (sSMC) 15 in Male Primary Infertility: A Case Study

**DOI:** 10.1155/carm/9935363

**Published:** 2025-04-23

**Authors:** Filomena Mottola, Renata Finelli, Veronica Feola, Kristian Leisegang, Lucia Rocco

**Affiliations:** ^1^Department of Environmental, Biological and Pharmaceutical Sciences and Technologies, University of Campania “Luigi Vanvitelli”, Caserta 81100, Italy; ^2^London Women's Clinic, London W1G6AP, UK; ^3^School of Natural Medicine, Faculty of Community and Health Sciences, University of Western Cape, Bellville 7535, South Africa

**Keywords:** infertility, small supernumerary marker chromosome, sperm aneuploidy, sperm DNA fragmentation, sperm parameters

## Abstract

This case report describes a 39-year-old phenotypically normal male patient of a married couple with primary infertility presenting as candidates for assisted reproductive techniques. The medical history of the couple is unremarkable, with both partners phenotypically normal. Semen analysis revealed oligoasthenzoospermia (OAT), 15% sperm DNA fragmentation and 4% aneuploidies in the sperm nuclei. Genetic analysis showed no Y chromosome of cystic fibrosis transmembrane conductance regulator gene mutations. Karyotype analysis in the male partner revealed a small supernumerary marker chromosome (sSMC) derived from chromosome 15, specifically inverted and duplicated (inv dup(15)) corresponding to the 15q11.2 region but lacking the Prader-Willi/Angelman syndrome critical region (PWACR). Further investigations revealed that 35% of the patient's spermatozoa carried the sSMC(15). This case study highlights the potential association between the presence of an inv dup(15) sSMC, without the involvement of the PWACR, and male infertility. sSMC(15) may disrupt spermatogenesis and contribute to oligoasthenozoospermia in males with primary infertility. Further research into the association of mechanism mechanisms of male infertility related to the 15q11.2 region is warranted.

## 1. Introduction

Small supernumerary marker chromosomes (sSMCs) are additional chromosomes to the normal diploid number that are typically small in size and structurally abnormal [1]. A higher incidence of sSMCs are observed in patients with a range of phenotypic abnormalities such as congenital anomalies, intellectual disabilities, and infertility [2]. Furthermore, males are affected 7.5 times more frequently than females [1]. A cooperative study involving 19 Italian laboratories, conducted in 2005, found that sSMCs occur at a rate of 0.18–1.5 per 1000 individuals in the general population, with significantly higher rates observed in patients with infertility [3]. Advanced cytogenetic techniques such as fluorescence in situ hybridization (FISH) and GTG banding are required for diagnostic detection of sSMCs [1].

sSMCs are most commonly derived from human chromosome 15, representing 25%–30% of cases. To date, the total number of reported sSMCs(15) is 1307, of which 32.44% represent cases without evident clinical findings, 36.04% have clinical findings, and the remaining include subjects with inverted and duplicated (inv dup(15)) and autism, cases with complex sSMCs, and those with uniparental disomy (UPD) [4]. Among sSMCs (15), several reported cases are associated with neuropsychiatric and behavioral disorders and involve the inv dup(15) [1]. Indeed, of the 1307 currently known cases of sSMCs(15), 117 are inv dup, commonly associated with autism or seizures [4] Rearrangements of chromosome 15 are associated with Prader-Willi Syndrome (PWS) or Angelman Syndrome (AS), depending on the paternal (PWS) or maternal (AS) deletion, disruption, duplication or triplication of the Prader-Willi/Angelman syndrome critical region (PWACR) [5]. In PWS, some maternal genes (including SNRPN) are silenced due to imprinting while paternal copies are generally deleted, and the lack of paternal contribution or presence of triplicated genes in the 15q11-q13 region leads to clinically apparent cognitive and behavioral defects [5]. However, sSMCs(15) usually do not involve the more distal PWACR of paternal or maternal origin, and these are generally considered phenotypically irrelevant for PWS and AS.

sSMCs(15) have been reported in infertile males, suggesting that heterochromatin in sSMCs is responsible for altering meiosis and consequently normal spermatogenesis [6]. However, the correlation between the presence of sSMCs(15) and male infertility is complex and poorly understood, particularly where no specific clinical signs are apparent in these patients. This case study reports a married male patient with primary infertility showing altered chromosomal arrangement on chromosome 15 due to the presence of a supernumerary marker, with no other contributing factors to infertility.

## 2. Case Presentation

A married couple with primary infertility presented at the Reproduction Biology Laboratory (University of Campania “Luigi Vanvitelli”) for genetic screening as candidates for assisted reproductive technology (ART) in October 2019. All procedures adhered to the ethical standards with the Helsinki Declaration of 1964 and its later amendments. The patient underwent routine analysis at the Reproduction Biology Laboratory for diagnostic purposes, and data was obtained retrospectively from the patient's records. Consent to publish was obtained from the partecipants. Personal information has been fully anonymized to ensure privacy. The patient was not actively recruited for the study and there is no potential for patient identification with the data provided.

Both the male partner (39 years old) and the female partner (34 years old) were phenotypically normal, with no significant family history of infertility, congenital abnormalities, or intellectual disabilities. The male partner had a sister with 2 children and no history of fertility or other medical concerns. Furthermore, there were no remarkable clinical features observed on history or physical examination of the male partner.

The male partner underwent a standard semen analysis, sperm DNA fragmentation using TUNEL assay and sperm aneuploidies analysis using FISH with 13, 18, 21, X and Y probes. Genetic screening was done for Y chromosome microdeletions and cystic fibrosis transmembrane conductance regulator (CFTR) gene mutations as possible contributing factors to infertility. Semen analysis revealed oligoasthenozoospermia, 15% sperm DNA fragmentation and 4% aneuploidies in the sperm nuclei (Table 1). No Y chromosome microdeletions or CFTR gene mutations were observed.

**Figure 1:**
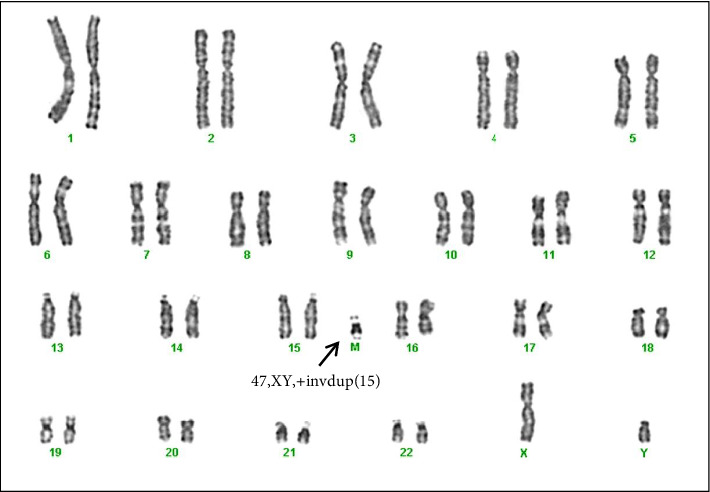
G-banding of the patient under examination, showing the chromosomal karyotype 47, XY, +mar. The arrow indicates the supernumerary marker.

Both partners subsequently underwent karyotype investigation on peripheral blood lymphocyte cultures by GTG banding for structural anomalies and aneuploidies. While normal karyotype was observed in the female, an additional chromosomal marker in the male partner was detected (Figure 1). The male partner karyotype was further investigated to establish the heterochromatic content using CBG staining and the presence of satellites using Ag nucleolus organizer regions (NOR) staining. In addition, investigation for various regions of chromosome 15 to confirm its origin were done, in particular bacterial artificial chromosomes (BACs) selected using the UCSC Genome Browser mapping in the 15q11.2 (RP11-11H9 and RP11-720B15), 15q12 (RP11-570N16) and 15q13.1 (RP11-408F10) regions.

**Figure 2:**
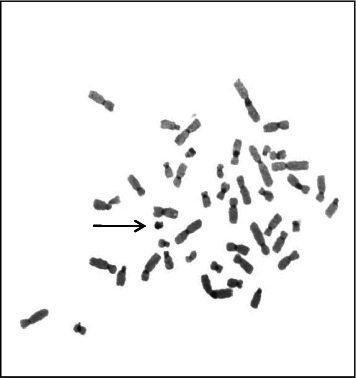
Chromosomal marker highlighted by C banding.

**Figure 3:**
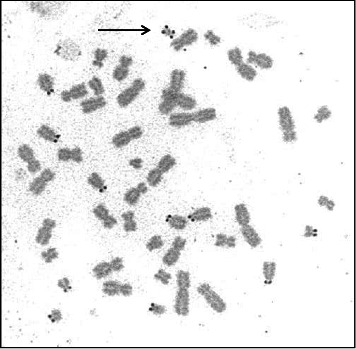
Satellite regions of chromosome 15 highlighted by Ag-NOR staining region (RP11-408F10) was detected (Figures 4(d) and 4(e)).

**Figure 4:**
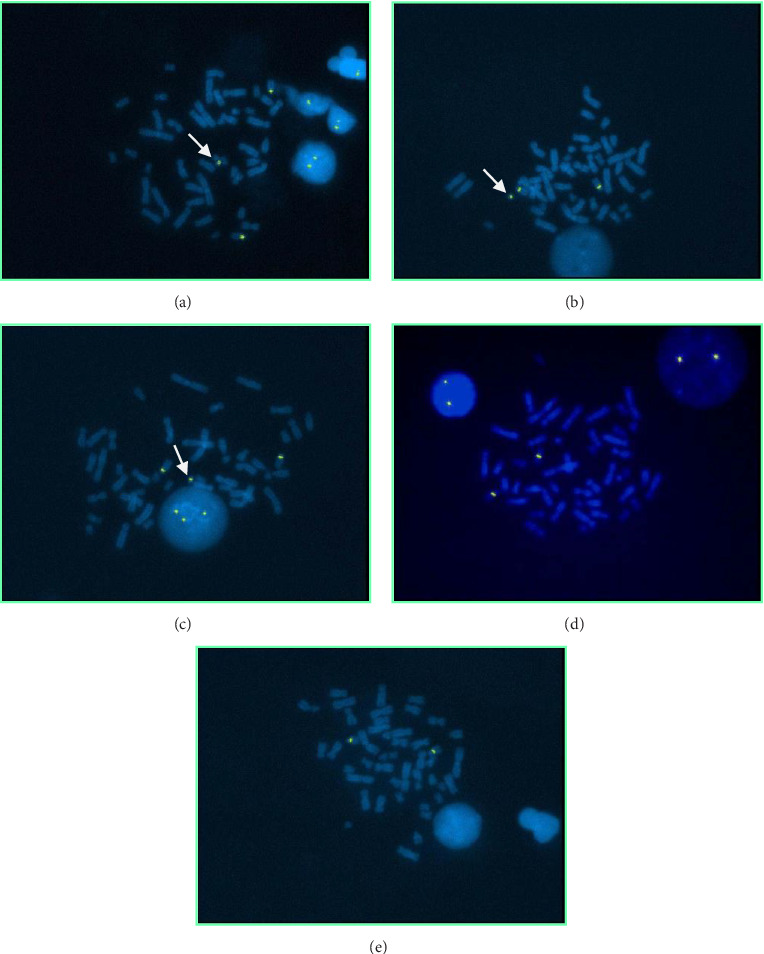
FISH on lymphocyte metaphases: (a) with D15Z1 probe; (b) with RP11-11H9 probe; (c) with RP11-720B15 probe; (d) with RP11-570N16 probe; (e) with RP11-408F10. In green, the hybridization signals of the various probes. Arrows indicate hybridization of the probe to the chromosome.

**Figure 5:**
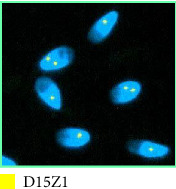
Sperm FISH with D15Z1 probe. In yellow, the hybridization signals of the probe marker.

A sSMC(15) with an intense C-banding staining was identified (Figure 2), which was inv dup(15) as indicated by the presence of four NOR regions corresponding to the 15q11.2 region (Figure 3). This detection was confirmed through two hybridization signals, one for the region immediately below the centromere of chromosome 15 (D15Z1) and another for the 15q11.2 region (RP11-11H9 and RP11-720B15). These signals were observed on both chromosome 15 and two on the marker chromosome, resulting in partial tetrasomy for the chromosomal region 15q11.2, therefore confirming the inv dup(15) marker (Figures 4(a), 4(b), and 4(c)). However, no probe signals were detected for the 15q12 (RP11-570N16) or the 15q13.1 regions. FISH analysis on spermatozoa covering the chromosome 15 region D15Z1 found that 35% of spermatozoa presented with the sSMC(15) (Figure 5).

## 3. Discussion

This case history presents a 39-year-old male partner of an infertile couple that showed oligoasthenozoospermia and relatively low levels of sperm DNA fragmentation and aneuploidies. Karyotype analysis in the male partner revealed a sSMC(15) characterized by an inv dup(15) corresponding to the 15q11.2 region.

sSMC inv dup(15) can present extremely variable clinical manifestations, influenced both by genetic mosaicism and the genetic content of the anomaly that could mitigate the clinical manifestations [7]. To more precisely identify the size of the sSMC in this case, the BACs RP11-11H9 and RP11-720B15 were used.

The BAC RP11-11H9 has a dual localization in the human genome according to UCSC (chr15:21056023-21056441 and chr15:22300187-22300706) [8], while the BAC RP11-720B15 is located within the range chr15:25356986-25569473, corresponding to the dosage-sensitive region on chromosome 15 [9].

The absence of a critical phenotype in the subject might indicate belonging to a subgroup of sSMC carriers without evident syndromes, as described in the ChromosOmics database, which includes similar cases [7, 10]. This discrepancy from the expected clinical outcomes may be explained by the presence of genetic mosaicism, often associated with a reduced clinical impact compared to what is normally anticipated. Unfortunately, a multi-tissue analysis, which could have provided more detailed information on the distribution of mosaicism and its actual correlation with the absence of clinical manifestations, was not performed. The diagnosis was based exclusively on blood analysis, limiting the ability to exclude with certainty the presence of mosaicism in other tissues, which could influence the subject's overall phenotype. However, infertility with OAT represents an important clinical manifestation associated with this condition, which, although not a clear critical syndrome typically associated with sSMC, contributes to defining the subject's clinical profile.

Previously, an inv dup(15) in the 15q11.2 region was reported in a 35 year old male presenting with azoospermia and a history of spontaneous abortions [6]. Similarly, a 38 year old male patient with oligoasthenoteratozoospermia presented an inv dup(15) in the 15q11.2 region, with no other abnormalities other than infertility [2].

Three different molecular sizes of inv dup(15) have been previously described [11]. The small size (type 1) includes only the centromeric locus D15Z1, the intermediate size (type 2) includes D15Z1 and D15S18, and the large size (type 3) is includes two additional copies of loci spanning D15Z1 to D15S12. Two regions on chromosome 15 are prone to instability and genomic breakage due to these repeating DNA elements. The region proximal to the centromere produces type 1 and type 2 inv dup(15) without encompassing the PWACR and without typical clinical features of PWS or AS, whereas the more distal region creates a type 3 inv dup(15) which includes the PWACR and clinical features of PWS or AS. In this case study, the chromosomal marker in the male partner was lacking the PWACR. However, the effect that the presence of an inv dup(15) has on spermatogenesis and meiotic segregation or rearrangement of normal chromosomes 15 is not fully understood.

Increased aneuploidy in sSMC carrier spermatozoa has been previously reported in 2 male patients compared to controls [12]. Furthermore, sSMC(15) with oligoasthenoteratozoospermia had a 7-fold higher level of autosomal disomy in a 30 year old male patient compared to sSMC negative males, with no significant effect on sperm chromatin integrity [13]. Similarly, a significant increase in the percentage of sperm with disomy of chromosomes X, Y and 18 compared to a fertile brother has been reported [14]. Therefore, the presence of sSMC(15) may disrupt spermatogenesis, contributing to oligoasthenozoospermia and sperm aneuploidy in this case.

There is significant variability in chromosomes and sSMCs segregation during meiosis, which depends on the sSMC origin and magnitude [15]. This supports the hypothesis that spermatogenesis may be affected when the presence of sSMCs in spermatozoa is relatively low. Furthermore, chromosome 15 is physically close to the XY pair during meiosis, where the sSMC would be attracted to its homologous sister chromosome and disturbing the formation of the synapsis between the adjacent sexual chromosomes [16]. This might lead to a meiotic arrest and consequently male infertility.

Previously, 37% and 30% of sperm nuclei presenting sSMC(15) in two cases of infertile males with sSMCs corresponding to the proximal 15q11.2 region have been reported [14]. Similarly, this case study found a relatively low level of spermatozoa (35%) with sSMC(15). There may be a possible selection against the genetic marker during spermatogenesis that contributes to the lower levels of sperm aneuploidy and DNA fragmentation observed in the patient, suggesting the presence of the sSMC(15) could induce interchromosomal effects such as non-disjunction or marker selection. The lower frequency of sperm monosomy or disomy, the low percentage of sperm DNA fragmentation and the deviation from the expected 1:1 segregation could be due to upstream apoptosis of chromosomally unbalanced sperm cells regulated by meiotic checkpoints. This process would eliminate sperm with altered DNA and further reduce sperm through triggering apoptosis. However, some genetically altered cells can escape the control mechanisms, contributing to oligozoospermia and azoospermia observed in patients with sSMC(15).

There are 18 genes that are expressed in the 15q11.2 region of the testis, including *POTEB* (POTE ankyrin domain family member B) gene family that encodes proapoptotic proteins. A high and specific expression of the POTE protein in primary spermatocytes may be responsible for the induction of apoptosis [14]. Therefore, abnormal expression of POTE due to its duplication could explain the oligoasthenozoospermia observed in this case study.

Limitations of this study outcomes include the retrospective nature and the reporting of a single case without targeted recruitment, which may not be representative of the broader population. While the study identifies the presence of sSMC(15) and its potential impact on infertility, the precise molecular mechanisms remain unclear.

In conclusion, this case study demonstrated that the presence of a sSMC(15) without the involvement of the PWACR may be associated with male infertility. Further investigations of the genes in the 15q11.2 region may contribute to the understanding of ‘the molecular mechanisms of male infertility. Patients with sSMCs may benefit from genetic counselling before assisted reproduction as potential transmission of genetic defects to the offspring remains a major concern.

## Figures and Tables

**Table 1:** Patient results and reference ranges for semen analysis.

Parameter	Patient results	Reference range
Sperm concentration	10.5 × 10^6^/mL	≥ 15 × 10^6^/mL
Sperm progressive motility	20%	≥ 32%
Sperm normal morphology	4%	≥ 4%
Sperm vitality	85%	≥ 58%
Seminal leukocytospermia	< 1 × 10^6^/mL	< 1 × 10^6^/mL
Sperm DNA fragmentation	15%	< 30%
Sperm aneuploidy	4%	< 4.84%

## Data Availability

The data that support the findings of this study are available on request from the corresponding author. The data are not publicly available due to privacy or ethical restrictions.
